# Relationships among Sleep Time, Physical Activity Time, Screen Time, and Nutrition Literacy of Adolescents: A Cross-Sectional Study in Chongqing, China

**DOI:** 10.3390/nu16091314

**Published:** 2024-04-27

**Authors:** Qi Xu, Zhichuan Hu, Mao Zeng, Yu Su, Ke Jiang, Shengping Li, Zhourong Li, Lin Fu, Zumin Shi, Manoj Sharma, Yong Zhao

**Affiliations:** 1 School of Public Health, Chongqing Medical University, Chongqing 400016, China; xuqi@stu.cqmu.edu.cn (Q.X.); 2021120768@stu.cqmu.edu.cn (Z.H.); 2021110631@stu.cqmu.edu.cn (K.J.); 2021120781@stu.cqmu.edu.cn (Z.L.); 2023121648@stu.cqmu.edu.cn (L.F.); 2 Research Center for Medicine and Social Development, Chongqing Medical University, Chongqing 400016, China; 3 Research Center for Public Health Security, Chongqing Medical University, Chongqing 400016, China; 4 Nutrition Innovation Platform-Sichuan and Chongqing, Chongqing 400016, China; 5 Department of Communicable Disease Control and Prevention, Chengdu Shuangliu District Disease Prevention and Control Center, Chengdu 610202, China; zengmao@stu.cqmu.edu.cn; 6 Chongqing Jiulongpo District Center for Disease Control and Prevention, Chongqing 400080, China; 15523433435@163.com; 7 Chongqing Health Center for Women and Children, Chongqing 400012, China; 15223329854@163.com; 8 Human Nutrition Department, College of Health Sciences, QU Health, Qatar University, Doha P.O. Box 2713, Qatar; zumin@qu.edu.qa; 9 Department of Social and Behavioral Health, School of Public Health, University of Nevada, Las Vegas, NV 89106, USA; manoj.sharma@unlv.edu; 10 Department of Internal Medicine, Kirk Kerkorian School of Medicine, University of Nevada, Las Vegas, NV 89106, USA; 11 Chongqing Key Laboratory of Child Nutrition and Heath, Children’s Hospital of Chongqing Medical University, Chongqing 400014, China

**Keywords:** nutrition literacy, sleep time, physical activity, screen time, adolescent, Chongqing

## Abstract

**Background:** Unhealthy lifestyles among adolescents are reaching alarming levels and have become a major public health problem. This study aimed to assess the relationship between sleep time, physical activity (PA) time, screen time (ST), and nutritional literacy (NL). **Methods:** This cross-sectional online study involving adolescents aged 10–18 years was conducted in September 2020 in 239 schools in Chongqing, China. NL was measured using the “Nutrition Literacy Scale for middle school students in Chongqing (CM-NLS)”. According to the recommended by the Chinese dietary guidelines (2022), we divided the sleep time of junior high school students into <9 h and ≥9 h, high school students into <8 h and ≥8 h, divided the workdays into weekend PA time < 1 h and ≥1 h, and divided the workdays into weekend ST < 2 h and ≥2 h. The multinomial logistic regression model was used to examine the association. **Results:** A total of 18,660 adolescents (50.2% males) were included. The proportion of participants that were junior high school students and attended boarding schools was 57.2% and 65.3%, respectively. Compared with senior high school students, junior high school students had a higher level of NL. Whether on workdays or weekends, participants with sleep time ≥ 8/9 h, PA time ≥ 1 h, and ST < 2 h per day had higher levels of NL. On weekdays, participants who met the sleep time ≥ 8 h/9 h (OR = 1.48, 95% CI: 1.36, 1.62) and PA time ≥ 1 h (OR = 1.69, 95% CI: 1.59, 1.81) had higher reporting of NL levels. **Conclusions:** Sleep time, PA time, and ST were positively correlated with NL among adolescents, especially junior high school students.

## 1. Introduction

Recent studies indicate a direct link between unhealthy behaviors in adolescence and increased risk of obesity [1], with significant impacts on future health [2,3], such as increased time spent in front of screens [4,5,6] and reduced physical activity [7,8] and sleep [2,9,10]. Furthermore, the literature shows a correlation between duration of physical activity and weight loss [11], and sedentary behaviors in youth predict high BMI in adulthood [3].

Multiple unhealthy lifestyle factors further exacerbate the risk of obesity, and adolescent obesity may increase the risk factor for cardiovascular disease morbidity and mortality in adulthood [12,13]. Therefore, healthy living habits and healthy behavior should be developed to prevent the occurrence of obesity in children and adolescents. Increasing evidence from epidemiological studies suggests that health literacy (HL) is significantly associated with obesity in adults and children [14,15,16]. HL knowledge and skills determine the consequential management of obesity in children and adults.

Nutrition literacy (NL), as a special HL, is defined as the capacity to obtain, process, and understand nutrition information and the materials needed to make appropriate decisions regarding one’s health [17,18]. Some results have suggested that adolescents with greater HL skills have healthier behavior, such as sleep time, PA time, and screen time (ST) [19,20]. However, few studies have been conducted on the relationship between NL and health behavior, and they mainly focused on the relationship between NL and eating habits [21].

Insufficient sleep time and ST have been proposed to be linked to unhealthy eating behavior [22,23]. People who exercise every day have better eating habits [24], and NL is the determinant of diet quality among young adolescents [25]. Previous studies on the relationship among sleep time, ST, PA time, and eating behavior did not explore NL. Thus, exploring health behavior (sleep time, PA time, and ST) and the NL status among adolescents and their relationships may help adopt effective strategies for promoting nutritional health among this critical age group. This research is expected to formulate reasonable sleep time, PA time, and ST for adolescents and provide strategies to improve the NL level of adolescents in China.

## 2. Materials and Methods

### 2.1. Study Design and Sample

This cross-sectional study was conducted in September 2020. Convenient sampling was adopted by randomly selecting 239 schools from 38 administrative districts in Chongqing. The domestic professional online survey platform “Questionnaire Star” was used to collect data. The questionnaire link or QR code was sent to the school health working group in each region through Chongqing Education Commission. The school health worker forwarded the questionnaire to the head teacher at the secondary school (grades 7, 8, 10, and 11), who instructed the students to fill out the questionnaire anonymously (Supplementary Table S1). Participants with dyslexia, inability to provide informed consent, endocrine disorders, or central nervous system damage diagnosed by a physician were excluded. This study was approved by the Ethics Committee of Chongqing Medical University (approval number: 2021041). All participants were informed about the study, and they provided consent prior to the survey.

### 2.2. Outcome Variable: NL Score

The “Nutrition Literacy Scale for Middle School Students in Chongqing (CM-NLS)” was developed by Zeng Mao et al. [26] in 2021 to measure the NL status of middle school students. NL mainly contains 52 items in three subdomains that are based on Nutbeam’s hierarchical model of HL, as described in detail by Wang et al. [27]. The scale has been tested for its validity (KMO = 0.916) and reliability (Cronbach’s ɑ of 0.849). Relative evaluation was adopted to convert the total NL score and each subdomain into the hundred-mark system. The higher the score was, the better the NL.

### 2.3. Exposure Variable: Health Behavior

Three different aspects of reported health behavior were included: sleep time, PA time, and ST. Sleep time included nap time. PA consists of housework, leisure activities, sports, mountain climbing, and rope skipping. ST refers to the time spent by middle school students in front of an electronic screen, such as a TV, computer, tablet, video game console, or mobile phone [28,29], during workdays and weekends.

As recommended by the Chinese dietary guidelines (2022), the sleep time of junior high school students was divided into <9 h and ≥9 h, and that of high school students was divided into <8 h and ≥8 h. Workdays were divided into weekend PA time < 1 h and ≥1 h, and workdays were divided into weekend ST < 2 h and ≥2 h [29].

### 2.4. Covariates

The following variables were considered covariates: age; gender (male or female), grade (students in grades 7 and 8 were in junior high school, and those in grades 10 and 11 were in senior high school); ethnicity (Han or minorities); body mass index (BMI), which was calculated using the formula weight in kg/(height in m)^^2^, classifying individuals as normal-weight, underweight, or overweight–obese according to WHO criteria [30]; boarding school (yes or no); residence (urban or rural); sibling (yes or no); caregiver model (parents or others); educational level (primary school and below, junior high school, high school/technical secondary school/vocational high school, or bachelor’s degree or above); and parents’ occupations. All data were self-reported.

### 2.5. Statistical Analysis

Data analysis was performed using STATA version 17 (STATA Corporation, College Station, TX, USA). The NL scores were calculated, and those above the median were categorized as high group, whereas those below the median were categorized as low group. Descriptive statistics include the frequency and percentage of studied variables. Chi-squared tests were conducted to describe the participants’ characteristics and assess the relationship among NL, health behavior, and covariates. A binary regression model was established with NL as the independent variable and health behavior as the dependent variable, which (sleep time < 8 h/9 h, PA time < 1 h, and ST ≥ 2 h) did not meet the guidelines’ recommendations that were used as reference. Dummy variables were created for each health behavior. Regression analyses were performed separately for each dummy variable. Odds ratios were adjusted for gender, grade, BMI, boarding school, residence, sibling, caregiver model, and educational level of parents. Statistical significance was considered when *p* < 0.05 (two-sided).

## 3. Results

### 3.1. Sample Description

Table 1 shows the demographic characteristics of the participants. A total of 18,660 middle school students were included in the study. Their mean age was 14.3 ± 1.8 years, 50.2% were male, and 27.3% were overweight or obese. More than half of the participants were junior high school students (57.2%) and attending boarding schools (65.3%). Junior high school students had a higher level of NL than senior high school students. Students who were not in boarding school had higher NL levels than their counterparts. Students raised by parents and those without siblings had higher NL levels than their counterparts. These results showed that the levels of NL varied among different educational levels of parents. Differences in NL were observed across all demographic characteristics except gender (*p* < 0.001).

### 3.2. Association between Health Behavior and NL

**Figure 1:**
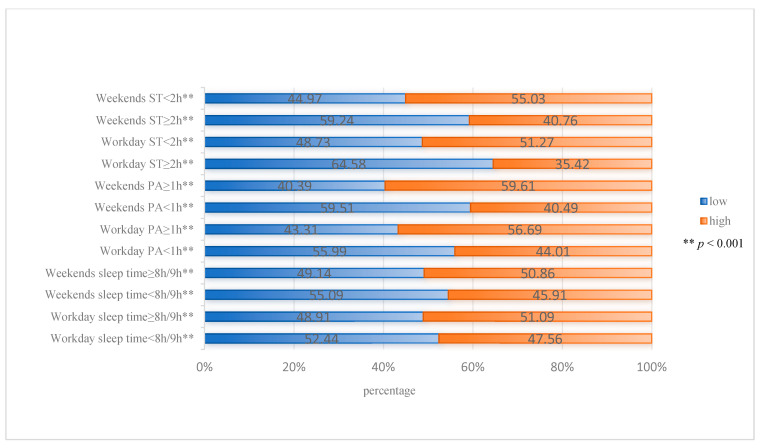
Distribution of nutritional literacy under different health behaviors.

The Chi-square test results of two-by-two comparison showed that different health behaviors had significant differences at the NL level (*p* < 0.001). Whether on workdays or weekends, participants with sleep time ≥ 8 h/9 h, PA time ≥ 1 h, and ST < 2 h per day had higher levels of NL (Figure 1).

According to the logistic regression results, whether on weekdays or weekends, sleep time ≥ 8 h/9 h, PA time ≥ 1 h, and ST < 2 h were positively correlated with high NL. Participants who had sleep time ≥ 8 h/9 h (OR = 1.48, 95% CI: 1.36, 1.62) and PA time ≥ 1 h (OR = 1.69, 95% CI: 1.59, 1.81) on weekdays demonstrated high NL levels after adjusting for demographic variables, including gender, grade, ethnicity, BMI, boarding school, place of residence, sibling, and caregiver pattern (*p* < 0.001). Similar results were found on weekends [31] (Table 2).

### 3.3. Subgroup Analyses of the Association between Health Behavior and NL

Whether on weekends or weekdays, an association was found between achieving the recommended sleep time, PA time, and ST and high NL across gender, grade, ethnicity, school boarding, sibling, and caregiver patterns (Table 3). Boys with sleep time ≥ 8 h/9 h on weekends exhibited higher NL level than girls. However, girls with ST < 2 h had a higher NL level. The results indicate that middle school students show higher levels of NL than high school students. Importantly, ST ≥ 8/9 h and PA time ≥1 h are associated with higher NL, regardless of the day of the week (*p* < 0.001).

Students who board at the school had a significant association with sleep time, PA time, and ST, with a statistically significant difference (*p* < 0.05). On weekdays, the NL level of students not living on campus and with sleep time ≥ 8 h/9 h or ST < 2 h was higher than those who live on campus.

## 4. Discussion

NL is considered to be a key determinant of an individual’s healthy eating habits and nutritional status, and it has a positive effect on an individual’s eating behavior [32,33]. In the present study, the relationship among sleep duration, PA time, ST, and NL in adolescents was investigated. This study shows a clear correlation between better sleep and physical activity habits and higher nutritional literacy among adolescents. These results are consistent with previous studies suggesting a positive impact of adequate sleep and regular physical activity on dietary choices and overall health status.

The results showed that grade, ethnicity, BMI, boarding school, residence, sibling, caregiver model, and educational level of parents were influencing factors of NL. Differences in NL levels were observed among adolescents of different grades and ethnicities, consistent with the conclusions of previous studies [34,35]. However, the proportion of high school students with high NL level was lower than that of middle school students, which is different from the conclusion of other studies [36], because high school students partake in a tight curriculum and rarely take nutrition and health courses [37]. The proportion of minority students with high NL levels was lower than that of Han nationality; this may be because ethnic minority students have fewer opportunities to receive nutrition and health education, and that minorities may have some unique dietary customs and unhealthy eating behavior [38]. Meanwhile, the NL levels of middle school students with different BMIs differed, contrary to the conclusions of previous studies [39,40]. People with poor NL may tend to consume more fried foods, sugary drinks, red meat, and processed foods, whereas people with better NL eat more vegetables, olive oil, and nuts [41]. People with different NL levels have different dietary structures, which also make them have different BMIs. The proportion of students with a high NL level in rural areas was lower than that in urban areas, which may be because students in urban areas have more opportunities to receive nutrition and health education; on the contrary, students in rural areas are affected by economic underdevelopment, poor implementation of basic life, limited access to nutrition information, and low awareness of developing good eating behavior [42].

This study found that the proportion of adolescents with a high NL level in parental care was higher than that in other types of caregiver models, and the proportion of students with higher parental education was higher. Research suggests that family environment may play an important role in adolescents’ eating behavior [43]. Living with grandparents was positively associated with child weight [44]. Studies have found that the functional NL of adolescents increases with the increase in fathers’ educational level [21]. Well-educated parents are better able to teach their children nutrition skills and have more resources to create opportunities for their children to learn and practice those skills [19,45]. They also are more likely to instruct them to eat more fruits and vegetables, consume less sweet drinks, and have more frequent PA time [46]. Strengthening parents’ health knowledge and capacity may help improve children’s health, especially in the areas of nutrition and health behavior. Improving the NL of children whose parents have a low educational level through a combination of parents’ education and community engagement interventions is critical to improve the overall well-being of these children.

Whether on weekdays or weekends, students with sleep time ≥ 8 h/9 h had a higher NL. Logistic regression showed that sleep time ≥ 8 h/9 h was positively correlated with NL, indicating that people with longer sleep duration had better NL. Studies have shown that sleep duration is positively correlated with fruit and vegetable intake and negatively correlated with sweets and snack intake and eating-out habits [47]. Short sleep time may lead to subtle changes in eating patterns that gradually alter the energy balance, thereby increasing the risk of obesity [48]. Short sleepers are more likely to switch from the traditional three meals a day to fewer main meals and consume energy-dense, highly palatable snacks more frequently during the night [49]. Therefore, adequate sleep has positive implications for maintaining healthy eating behavior, eating regularly, and maintaining a healthy weight.

PA time and good nutrition are important behavioral factors in promoting health and preventing disease [50]. Logistic regression showed that exercise time ≥ 1 h was positively correlated with NL, suggesting that people who exercised for a longer time had better NL. Studies have shown an association between higher nutritional knowledge and higher PA time [51]. Some studies have found a correlation between nutrition and exercise behavior and the HL level of adolescents [52], and HL is positively correlated with children’s PA time [53]. Students with lower HL reported a lower frequency of health-promoting behaviors, a higher frequency of risky health behaviors, and poorer quality of life than students with higher HL [52]. Therefore, people who exercise longer may have higher NL because they have better HL. It is recommended that schools should adapt their curricula to ensure that students have at least one hour of physical activity both within and outside of the school setting each day, as a means to enhance their nutritional status through improved HL.

From an adolescent perspective, the Internet is seen as a powerful resource in health information and usage [54] and a health hazard due to prolonged use of electronic products. Logistic regression showed that the people with ST < 2 h were positively correlated with NL, indicating that people with less ST had better NL. Grassi et al. [55] and Tambalis et al. [22] found similar results. Students who watched < 1 h of TV per day had significantly higher NL scores than those who watched more TV. This finding may be due to the increasing exposure of adolescents to the marketing of unhealthy foods, including eating videos, social media, and point-of-sale advertising [21]. Zimmerman FJd [51] suggested that advertisements and programs showing unhealthy food and learning about unhealthy nutritional habits may contribute to obesity. Meanwhile, increased ST is a risk factor for overweight/obesity among children and adolescents [56]. When the time spent watching TV increases, the frequency of snacking also increases. When watching TV, people tend to eat foods with low nutritional value and reduce the intake of fruits, vegetables, and milk [21]. This finding may be another explanation for the relationship between ST and NL, and the use of electronic products may lead to eating many foods with low nutritional value, leading to unhealthy eating behavior.

Subgroup analysis showed that gender, grade, ethnicity, residence, siblings, caregiver model for sleep time, PA time, and the relationship between ST and NL are influenced by different types, degrees, and times. In our study, boys were more likely than girls to sleep the recommended amount of time, especially on weekends. This may be due to the fact that girls tend to be “distracted” by other things, leading to late nights. Related studies have found that girls are more easily distracted by electronic devices and spend more time communicating with friends (boys and girls) via cell phones [57,58]. This suggests an underlying gender factor influencing sleep. The relationship between sleep and exercise times on weekdays and the NL of middle school students was stronger than that of high school students, and the relationship between PA time and ST on weekends was stronger than that of high school students. High school students may have fewer hours of sleep and physical activity on weekdays due to homework loads and earlier school days. Gunderson J’s [59] study found that more than three-quarters (78.4%) of students do not get eight hours or more of sleep on school nights. Our study found that high school students were less likely to be physically active for the recommended amount of time compared to middle school students. This is consistent with other studies that show a decline in physical activity with age [60,61]. In addition to this, students with a normal BMI are more likely to meet the recommended physical activity hours on weekends. Overweight/obese students are often sedentary and physically inactive [62]. Given the significant influence of academic workload on the time allocated to sleep and exercise among high school students, it is imperative that schools incorporate comprehensive health and nutrition education into the curriculum to mitigate potential declines in nutritional status.

Not residing in boarding schools had a greater effect on the relationship among sleep time, PA time, ST, and NL, and having no siblings had a greater effect on the relationship between sleep time and NL on weekdays. The relationship between PA time on working days and NL was more affected. Studies have shown that students who live on campus do not eat a sufficiently wide variety of meals [52]. Peer influence on healthy eating behavior in children and adolescents is often negative, leading them to consume more energy-dense foods with low nutritional value [53]. This finding could partly explain the findings of the present study, because live-in students and those without siblings were less likely to be influenced by their peers during non-school hours. For non-boarding school students and those without siblings, a targeted approach involving both schools and caregivers is necessary to promote a balanced lifestyle that includes adequate sleep, regular physical activity, and restricted screen time. Compared to students living in rural areas, living in towns is a protective factor for meeting recommended values for sleep time, physical activity time, and screen time. The higher the socio-economic status of a family, the healthier the lifestyle habits of its parents, which have a positive impact on their children. Students raised by their parents are more likely to meet the recommended values for sleep and physical activity on weekends. Educational measures to address unhealthy eating and physical activity behaviors should be developed in collaboration with parents.

In conclusion, based on the relevant guidelines and standards, and in conjunction with the results of this study, we recommend that adolescents should maintain 60 min of moderate-to-vigorous aerobic physical activity per day [63,64,65], get at least 8 h of sleep per day [63,66], and spend no more than 2 h per day on video screens [63,67].

## 5. Limitations

This study has some limitations. First, the researcher’s ability to draw direct causal inferences is compromised by the use of cross-sectional survey data, underscoring the importance of longitudinal studies for establishing causality and corroborating these findings in the future. In future studies, we should conduct a cohort study to explore the relationship between sleep time, PA time, ST, and NL. Second, although quality control was strictly enforced in the process, online surveys and self-reported surveys inevitably bring some information bias. Third, this study was conducted during the initial phase of the COVID-19 pandemic, when students may have been confined to their homes with limited physical activity resulting in decreased exercise time and increased screen time due to increased online classes or time spent playing with electronics.

## 6. Conclusions

In conclusion, the present study emphasizes the importance of promoting healthy behaviors, such as adequate sleep and regular physical activity, to improve nutritional literacy among adolescents. Future research should explore interventions aimed at integrating these healthy practices into students’ daily lives to combat obesity and promote healthier living.

## Figures and Tables

**Table 1:** Distribution by levels of nutrition literacy across demographic characteristics (*n* = 18,660).

Variables	All	NL		*p*-Value
	*N* (%)	Low (*n* = 9688)	High (*n* = 8972)	
Age (mean, SD)	14.3 (1.8)	14.6 (1.8)	13.9(1.7)	<0.001
Gender				0.074
Male	9359 (50.16)	4920 (52.57)	4439 (47.43)	
Female	9301 (49.84)	4768 (51.26)	4533 (48.74)	
Grade				<0.001
Junior high school	10,670 (57.18)	4615 (43.25)	6055 (56.75)	
High school	7990 (42.82)	5073 (63.49)	2917 (36.51)	
Ethnicity				<0.001
Han	16,581 (88.86)	8272 (49.89)	8309 (50.11)	
Minority	2079 (11.14)	1416 (68.11)	663 (31.89)	
BMI				<0.001
Normal	10,579 (56.69)	5327 (55.35)	5252 (49.65)	
Underweight	2986 (16.00)	1599 (53.55)	1387 (46.45)	
Overweight-obesity	5095 (27.31)	2762 (54.21)	2333 (45.79)	
Boarding school				<0.001
Yes	12,183 (65.29)	6886 (56.52)	5297 (43.48)	
No	6477 (34.71)	2802 (43.26)	3675 (56.74)	
Residence				<0.001
Urban	9062 (48.56)	4219 (46.56)	4843 (53.44)	
Rural	9598 (51.44)	5469 (56.98)	4129 (43.02)	
Siblings				<0.001
Yes	14,782 (79.22)	7879 (53.30)	6903 (46.70)	
No	3878 (20.78)	1809 (46.65)	2069 (53.35)	
Caregiver model				<0.001
Only parents	13,313 (71.35)	6766 (50.82)	6547 (49.18)	
Others	5347 (28.65)	2922 (54.65)	2425 (45.35)	
Father’s education				<0.001
Primary school and below	4176 (22.38)	2519 (60.32)	1657 (39.68)	
Junior high school	9539 (51.12)	4978 (52.19)	4561 (47.81)	
High school ^a^	3264 (17.49)	1479 (45.31)	1785 (54.89)	
Bachelor’s degree or above	1681 (9.01)	712 (42.36)	969 (57.64)	
Mother’s education				<0.001
Primary school and below	5852 (31.36)	3566 (60.94)	2286 (39.06)	
Junior high school	8555 (45.85)	4295 (50.20)	4260 (49.80)	
High school ^a^	2865 (15.35)	1267 (44.22)	1598 (55.78)	
Bachelor’s degree or above	1388 (7.4)	560 (40.35)	828 (59.65)	

^a^: High school includes high school/technical secondary school/vocational high school.

**Table 2:** Logistic regression model of health behavior and nutritional literacy OR (95% CI).

Variables		Model 1	Model 2
Workday	Sleep time (≥8 h/9 h)	1.15 (1.06, 1.25) *	1.46 (1.34, 1.59) **
	PA time (≥1 h)	1.67 (1.57, 1.77) **	1.69 (1.58, 1.80) **
	ST (<2 h)	1.92 (1.78, 2.07) **	1.56 (1.45, 1.69) **
Weekends	Sleep time (≥8 h/9 h)	1.22 (1.15, 1.29) **	1.44 (1.35, 1.53) **
	PA time (≥1 h)	2.17 (2.04, 2.30) **	2.10 (1.98, 2.24) **
	ST (<2 h)	1.78 (1.68, 1.88) **	1.66 (1.57, 1.77) **

Model 1: Not adjusted. Model 2: Demographic variables were adjusted for gender, grade, BMI, ethnicity, boarding school, place of residence, siblings, and caregiver pattern. * *p* < 0.05, ** *p* < 0.001.

**Table 3:** Subgroup analyses of the association between NL and health behaviors.

	Workdays						Weekends					
Variables	Sleep Time (≥8 h/9 h)	*p* ^a^	PA Time (≥1 h)	*p* ^a^	ST (<2 h)	*p* ^a^	Sleep Time (≥8 h/9 h)	*p* ^a^	PA Time (≥1 h)	*p* ^a^	ST (<2 h)	*p* ^a^
Gender		0.961		0.769		0.551		0.049		0.614		0.034
Male	1.48 (1.32–1.66) **		1.71 (1.56–1.87) **		1.53 (1.37–1.70) **		1.55 (1.42–1.69) **		2.09 (1.92–2.28) **		1.61 (1.48–1.75) **	
Female	1.45 (1.28–1.65) **		1.67 (1.52–1.84) **		1.60 (1.43–1.79) **		1.35 (1.24–1.47) **		2.13 (1.95–2.33) **		1.74 (1.60–1.90 **)	
Grade		0.000		0.000		0.908		0.430		0.000		0.000
Junior high school	1.73 (1.52–1.97) **		1.89 (1.74–2.06) **		1.55 (1.38–1.73) **		1.41 (1.30–1.53) **		2.37 (2.19–2.57) **		1.94 (1.79–2.09) **	
High school	1.28 (1.14–1.44) **		1.43 (1.29–1.59) **		1.57 (1.41–1.74) **		1.48 (1.35–1.62) **		1.78 (1.61–1.96) **		1.36 (1.24–1.50) **	
BMI		0.195		0.653		0.926		0.280		0.108		0.333
Normal	1.45 (1.30–1.63) **		1.67 (1.53–1.82) **		1.58 (1.43–1.76) **		1.39 (1.29–1.51) **		2.19 (2.01–2.37) **		1.64 (1.51–1.77) **	
Underweight	1.31 (1.05–1.63) *		1.77 (1.50–2.08) **		1.58 (1.30–1.91) **		1.43 (1.23–1.67) **		2.18 (1.87–2.55) **		1.81 (1.55–2.11) **	
Overweight	1.58 (1.34–1.85) **		1.68 (149–1.91) **		1.51 (1.30–1.75) **		1.55 (1.37–1.74) **		1.91 (1.70–2.15) **		1.64 (1.46–1.84) **	
Ethnicity		0.483		0.373		0.088		0.009		0.044		0.000
Han	1.45 (1.32–1.59) **		1.70 (1.59–1.82) **		1.60 (1.47–1.73) **		1.40 (1.32–1.50) **		2.15 (2.02–2.30) **		1.72 (1.61–1.83) **	
Minority	1.51 (1.21–1.88) **		1.62 (1.33–1.97) **		1.32 (1.06–1.64) *		1.79 (1.48–2.18) **		1.73 (1.42–2.10) **		1.28 (1.06–1.55) *	
Boarding school		0.006		0.009		0.006		0.452		0.000		0.009
Yes	1.37 (1.24–1.51) **		1.93 (1.72–2.16) **		1.46 (1.33–1.59) **		1.47 (1.36–1.59) **		1.93 (1.79–2.09) **		1.57 (1.46–1.70) **	
No	1.81 (1.52–2.15) **		1.58 (1.46–1.71) **		1.84 (1.59–2.14) **		1.39 (1.25–1.54) **		2.47 (2.22–2.74) **		1.86 (1.68–2.05) **	
Residence		0.161		0.667		0.167		0.802		0.493		0.007
Urban	1.56 (1.36–1.78) **		1.72 (1.56–1.89) **		1.66 (1.48–1.86) **		1.46 (1.34–1.59) **		2.18 (1.99–2.38) **		1.81 (1.66–1.97) **	
Rural	1.42 (1.27–1.59) **		1.66 (1.52–1.81) **		1.46 (1.33–1.63) **		1.42 (1.31–1.55) **		2.04 (1.87–2.22) **		1.54 (1.41–1.67) **	
Siblings		0.050		0.083		0.904		0.923		0.047		0.950
Yes	1.42 (1.29–1.55) **		1.65 (1.53–1.77) **		1.56 (1.43–1.70) **		1.44 (1.34–1.54) **		2.04 (1.90–2.19) **		1.66 (1.55–1.77) **	
No	1.76 (1.42–2.18) **		1.86 (1.61–2.15) **		1.55 (1.30–1.86) **		1.44 (1.26–1.65) **		2.37 (2.06–2.73) **		1.67 (1.46–1.90) **	
Caregiver model		0.282		0.995		0.793		0.015		0.663		0.042
Only parents	1.42 (1.28–1.58) **		1.69 (1.50–1.91) **		1.57 (1.43–1.72) **		1.52 (1.41–1.63) **		2.14 (1.98–2.30) **		1.61 (1.50–1.73) **	
Others	1.56 (1.34–1.82) **		1.68 (1.56–1.82) **		1.55 (1.34–1.78) **		1.27 (1.13–1.42) **		2.04 (1.98–2.30) **		1.82 (1.62–2.03) **	

Model adjustments included gender, grade, BMI, ethnicity, boarding school, place of residence, siblings, and caregiver patterns. ^a^ P for interaction. * *p* < 0.05,** *p* < 0.001.

## Data Availability

The datasets generated and/or analyzed during the current study are not publicly available due to funding requirements but are available from the corresponding author on reasonable request.

## Supplementary Materials

The following supporting information can be downloaded at: https://www.mdpi.com/article/10.3390/nu16091314/s1, Table S1: Questionnaire.
