# Supplementary material for: Relationships among Sleep Time, Physical Activity Time, Screen Time, and Nutrition Literacy of Adolescents: A Cross-Sectional Study in Chongqing, China
Source: Nutrients. 2024 Apr 27;16(9):1314. doi: 10.3390/nu16091314 (PMC11085315; doi:10.3390/nu16091314)
Supplement: Supplementary file 1 [file nutrients-16-01314-s001.zip › Chinese Nutrition Society - Nutrition science Popularization and communication research Foundation materials.pdf]

合同号：【】

中国营养学会-营养科普与传播研究基金
资助合同

甲方：中国营养学会（以下简称“甲方”）
地址：北京市朝阳区建国门外大街甲 14 号北京广播大厦 1405
邮编：100022
电话：010-83554781
传真：010-83554780

乙方：【重庆医科大学】
项目负责人：【赵勇】
身份证号：【51022519760620077X】
地址： 【重庆市渝中区医学院路 1 号重庆医科大学】
电话：【13883460842】
邮箱：【zhaoyongzb@qq.com】

“中国营养学会-营养科普与传播研究基金”（以下简称“基金”）作为中国营养学会营养科研基金之一，2020 年以鼓励传统优良饮食文化与落实合理膳食为目标，研究饮食习俗、文化对大众健康的作用和影响，是推进我国合理膳食、新健康饮食文化传承的专项公益基金。

经甲方营养科研基金管理委员会组织专家评审和基金管理委员会批准，【重庆医科大学】申请的项目【西南地区“吃得文明”量化评价与倡导对策研究】，项目编号：【CNS-SCP2020-34】，获得 2020 年度营养科普基金资助人民币（含税）【贰拾】万元整（小写：【20】万元 ）。项目执行时间为【2020】年【6】月【1】日起至【2021】年【6】月【30】日止。为确保受资助项目按期、按申请书和任务书实施，双方特签订以下合同，以资共同遵守：

一、双方的权利和义务

1. 甲方按本合同约定向乙方拨付项目资助资金；乙方须按甲方批准的《中国营养学

扫描全能王 创建

会-营养科普与传播研究基金项目申请书》填写课题任务书，并按计划完成研究内容和考核指标。

2. 乙方应保证：乙方申报项目的真实性、合法性，并保证按计划使用所获资助资金，符合国家相关法律法规规定，专款专用，且实际用途不得偏离申报项目时的既定用途。

3. 乙方可以书面形式对项目资助资金数额、用途等向甲方提出更改要求，甲方对乙方的书面要求进行审核并书面同意后才能更改项目资助资金数额、用途。

4. 甲方有权检查项目进度，并予以督促和管理；甲方认为必要时，有权检查乙方的实验记录和实验结果，乙方应服从甲方的管理并配合。

5. 乙方应按甲方基金项目管理要求，及时、定期向甲方提交项目中期报告、结题报告以及相关的有效产出原始数据资料等，并保证提交给甲方的报告、数据、资料的真实性、合法性、完整性、有效性。按照中国营养学会营养科普与传播研究基金管理要求，乙方应在项目中期（以整个项目期限中间点为提交时间）向甲方提交中期进展报告；在项目规定结题时限提前 60 个工作日，应向甲方提交完整的、有原始数据的《结题报告》，作为项目完成的证明文件；研究项目验收合格后一年内，乙方须按照项目书要求将研究产出成果（包括不仅限于发表文章、专利申请，人才培养等）和其他相关材料，以电子版形式提交到中国营养学会科普与传播部（邮箱：kp@cnsoc.org）。

6. 乙方须严格按照申请书中计划进度和研究内容，如期在项目期限内完成项目研究任务。如遇特殊情况需延长项目执行时间，需提前 90 个工作日向甲方基金管理委员会提交书面申请，经基金管理委员会书面批复同意后，方可适当延期。

7. 乙方由甲方基金资助项目产生的相关成果（包括但不限于文章、专著等），必须严格按本合同约定在发表、宣传、宣讲、转载等载体上明确标注：“中国营养学会-营养科普与传播研究基金资助项目”，并注明项目编号。

8. 乙方应要求乙方工作单位为其建立专用账户，专款专用、决算实事求是。乙方工作单位负有经费使用的管理责任。甲方有权对乙方经费的使用、管理进行监督，包括但不限于引进第三方审计机构对乙方及乙方工作单位的经费使用管理情况进行专项审计。

9. 乙方在项目执行期间，因特殊原因不能继续负责该项目时，应提前【30】个工作日以书面形式上报甲方基金管理办公室，并提出延期或更换负责人的申请。甲方基金管理办公室在收到申请 10 个工作日内将基金管理委员会关于项目处理决议（例如：更换负责

扫描全能王 创建

人、继续执行或终止研究项目等）以书面形式通知乙方。对于终止研究的项目，乙方应退还尚未使用的项目资助资金，且甲方不再继续拨付未拨付的费用。

10. 未经甲方书面同意，乙方不得将本合同项目部分或全部研究开发工作转让给第三方承担。乙方如需将本合同项目部分或全部研究开发工作转委托、转包或分包给第三方的，应以书面形式向甲方提出并获得甲方同意，此时相关费用可由甲方直接支付给该第三方。

二、项目资助资金的支付

1. 本资助资金分两次支付。本合同生效后 30 个工作日内，甲方通过中国营养学会-科普与传播研究基金专用账户向乙方指定的账户支付该项目首笔资助款，即项目资助资金总金额的 70%，即人民币【壹拾肆】万元整（小写：【140000】）；项目预验收合格（验收合格标准为：提交完整结题报告，完成项目任务书中规定的研究目标、考核指标，提交论文、专利申请文件、著作等项目成果证明文件，并经甲方书面认可）后 30 个工作日内，甲方向乙方账户支付剩余资助款，即项目资助资金总金额的 30%，即人民币【陆】万元整（小写：【60000】）。

2. 甲方每次付款前，乙方所在单位应向甲方提供等额有效**增值税普通发票**；若乙方未在甲方付款前提供等额有效增值税普通发票，甲方有权顺延或拒绝付款且不承担违约责任。

乙方指定收款账户信息如下：

获资助项目依托单位银行账户信息：

收款单位：【重庆医科大学】

开户行：【中国建设银行重庆九龙坡金凤支行】

银行帐号：【50001033600050008726】

乙方上述收款信息如有变更，应及时以书面形式通知甲方；否则由此导致的不利后果，由乙方自行承担。

甲方开票信息如下：

名称：【中国营养学会】

扫描全能王 创建

税号:【51100000500002379R】

地址:【北京市宣武区广内大街6号枫桦豪景A座5单元1601、1602室】

电话:【010-83554781】

开户银行:【北京银行宣武门支行】

账号:【01090511100120109050884】

三、知识产权归属

1. 受资助项目的研究成果的知识产权(包括但不限于专利、著作权)及其衍生权利(包括但不限于商业开发权、收益权)均归双方永久性全部和完整所有,甲方及甲方的关联方均有权【永久性免费使用乙方研究结果的知识产权】,并有权就乙方的研究成果进行宣传推广。一方转让或授权许可其研究成果(包括但不限于根据研究成果形成的产品或专利技术)时,未经另一方事先书面同意,不能将研究成果(包括但不限于根据研究成果形成的产品或专利技术)转让、授权许可给与甲方或其关联方有竞争关系的第三方。

2. 乙方签订本合同后,被视为同意甲方及其关联方永久性免费在任何媒体(包括但不限于新闻媒体、自媒体等)以公益目的使用乙方姓名、肖像及乙方承担的该基金项目研究工作及其成果。甲方及关联方开展与该项基金资助项目相关宣传时,乙方有义务及时提供相关材料,并及时配合甲方对宣传资料学术内容的科学性、准确性进行复核。

3. 乙方在任何场合发布"中国营养学会-营养科普与传播研究基金"资助项目的工作时有责任阐明基金对其的资助,并将该次发布的资料副本于发布之日起【10】个工作日内送交甲方基金管理委员会。乙方在进行前述发布时,不得做出任何引人误解或引起混淆的行为,使他人误以为其是甲方或/及甲方关联方之工作人员或与甲方或/及甲方关联方具有其他实质性关系。

4. 未经甲方事先书面许可,乙方不得擅自使用、复制甲方及/或甲方关联方的名称、商标、商号、产品、域名、网站名称、标志或其他任何甲方及/或甲方关联方拥有知识产权的内容。

5. 甲方为本合同所述目的向乙方提供的所有技术资料、图纸、规范、手册、文件、数据及其他信息的所有权和其他知识产权归甲方所有。甲方履行本合同不得被理解为甲方

扫描全能王 创建

以明示或默示的方式将甲方拥有的知识产权转让或许可给乙方。

6. 乙方保证其完成的项目研究成果不违反法律法规规定，不侵害任何第三方包括但不限于知识产权、肖像权、隐私权在内的任何权利。如甲方接到第三方对乙方提交的项目研究成果侵权的通知，或者因乙方的项目成果而收到法院、仲裁机构等的传票/仲裁通知，甲方有权暂停本合同的履行，甲方应及时通知乙方，乙方应在收到甲方通知之日起七个工作日内与第三方协商解决。如经法院或仲裁机构判决、裁决、或与他人和解、调解导致甲方因前述事宜承担赔偿/补偿责任的，则乙方应赔偿甲方因此所受的全部损失，包括但不限于经法院或仲裁机构判决、裁决、或与他人和解、调解导致甲方因前述事宜承担赔偿/补偿费用以及甲方支出的律师费、诉讼费、差旅费等。

四、保密

1. 未经对方事先书面许可，一方不得向第三方（有关法律、法规、政府部门、证券交易所或其他监管机构要求和双方的法律、会计顾问、授权雇员除外）泄露本合同的条款的任何内容以及本合同的签订及履行情况，以及通过签订和履行本合同而获知的对方及其关联方的任何信息，也不得擅自自行使用。

2. 接收上述信息的一方保证，仅为履行本合同之目的而复制和使用对方提供的信息，并仅向其确有知悉必要的人员披露对方提供的信息，同时保证该人员接收、使用上述信息时，同样遵守本合同约定的保密义务。

3. 本合同失效、被撤销、解除或终止时，或者甲方要求返还其提供的保密信息时，乙方应按照甲方要求按时返还其已收到的保密信息的全部文件、资料及其全部复印、复制、誊写、摘要文件，如无法返还或返还无意义的，应按照甲方要求采取删除、销毁等措施并向对方提供删除、销毁的证明材料。

4. 本保密义务不受本合同期限的限制，不因本合同的解除、终止、无效、被撤销而失效。

五、合同的变更、解除和终止

1. 本合同生效后，任何一方欲变更协议内容的，应协商一致后，签订补充协议。但

扫描全能王 创建

因法律法规、政策、不可抗力等原因导致本合同具体条款履行需变更的，本着诚信合作、实现合同目的的精神，双方应当友好协商对协议进行补充、变更约定。

2. 除本合同另有约定外，乙方无权单方解除本合同。

3. 一方主体资格消失（包括但不限于破产、注销、死亡等）的，本合同自该方主体资格消失之日起自动终止。

4. 如发生情势变化，可能影响本合同的履行或协议目的的实现，则甲方可以提前五日书面通知乙方后即可单方解除本合同，则本合同自动终止。

六、违约责任

1. 如乙方存在下列情形之一的，甲方有权剥夺乙方“中国营养学会-营养科普与传播研究基金资助项目”资格，并单方解除本合同，并有权要求乙方退还已获得的项目资金，若甲方因此遭受任何损失（包括但不限于负面舆论、声誉损失、经济损失）的，甲方有权要求乙方赔偿全部损失：

1）乙方申报项目为虚假或不合法或不合规，或未按计划使用所获资助资金，实际用途偏离申报项目时的既定用途的；

2）乙方发表甲方基金资助项目产生的相关成果（包括但不限于文章、专著等）时，未在发表、宣传、宣讲、转载等载体上明确标注：“中国营养学会-营养科普与传播研究基金资助项目”及项目编号，且在甲方要求限期更正时，拒绝或怠于更正的；

3）乙方未通知甲方及甲方关联方的情况下，转让或授权许可其研究成果（包括但不限于根据研究成果形成的产品或专利技术），导致甲方及甲方关联方无法行使优先受让、优先被许可权的；

4）乙方未经甲方事先书面同意，擅自将研究成果（包括但不限于根据研究成果形成的产品或专利技术）转让、授权许可给与甲方或其关联方有竞争关系的第三方的；

5）乙方未按申请书及任务书的内容、技术路线完成研究目标与任务，或研究质量不佳，经甲方提醒、督促仍不能及改正或改进的；

扫描全能王 创建

6）乙方未按甲方基金管理要求如期报送项目《进展(中期)报告》和《结题报告》，无正当理由拖延项目实施，经甲方书面催办仍无法立即实施的；

7）项目实施过程中，乙方存在经费使用不当或学术不端或品行不端或违法犯罪等行为的。

2. 任何一方违反知识产权条款、保密条款的，应向对方支付本合同金额的 50%作为违约金，如违约金不足以弥补对方因此遭致的损失的，违约方应继续赔偿对方因此遭致的全部损失。

3. 除本合同另有约定外，任何一方直接或间接违反本合同的任何条款，或不承担或不及时、充分地承担本合同项下其应承担的义务即构成违约，守约方有权以书面通知要求违约方纠正其违约行为并采取充分、有效、及时的措施消除违约后果，并赔偿守约方因违约方之违约行为而遭致的损失。若违约方在收到守约方关于其违约行为的上述通知后三个工作日内未纠正其违约行为，守约方有权在做出书面通知情况下提前解除本合同。

4. 除本合同另有约定外，在违约事实发生以后，经守约方的合理及客观的判断，该等违约事实已造成守约方签署本合同的目的实现从根本上成为不可能，则守约方有权在做出书面通知情况下提前解除本合同，违约方应赔偿守约方因违约方之违约行为而遭致的所有直接损失。

七、不可抗力

1. “不可抗力”是指双方不能合理控制、不可预见或即使预见亦无法避免的事件，该事件妨碍、影响或延误任何一方根据协议履行其全部或部分义务。该事件包括但不限于政府行为、自然灾害、战争、传染病、罢工、法律或政策的变化或任何其他类似事件。

2. 出现不可抗力事件时，受影响方应及时、充分地以书面形式通知对方，并告知对方该类事件对本合同可能产生的影响，并应当在不可抗力事件发生后 5 日内提供该等事件的详细信息及由有关组织出具的解释受影响方因此无法履行全部或部分本合同项下义务的相关证明。

3. 由于以上所述不可抗力事件致使协议的部分或全部不能履行或延迟履行，则双方于彼此间不承担任何违约责任，但双方应尽力减少不可抗力对本合同的影响，并采取合理

扫描全能王 创建

的方式避免损失的扩大。

4. 不可抗力事件终止或被排除后，对方要求继续履行合同的，受影响方应继续履行，但受影响方有权要求延长履行义务的时间，延长期应相当于不可抗力事件实际造成延误的时间；如双方无法就恢复或延长合同的履行期限达成一致意见的，可以协商解除合同，此时在合同项下具有付款义务的一方不再支付未履行部分的款项，已支付的，收款方应予退还。

5. 不可抗力事件持续 30 天以上的，则任何一方有权以书面通知对方的方式单方解除本合同。

八、通知和送达

1. 本合同的一方发给另一方的任何通知、文件或申请均应以书面形式按照本合同页首列明的联系方式通过特快专递、电子邮件等形式发出。如果以特快专递的方式发出的，则在邮寄后第 5 日视为送达；如果以电子邮件方式发出的，以发出方系统显示电子邮件成功发送时视为送达。

2. 本合同记载的通讯地址、联系方式发生变更的，应在变更后 2 日内书面通知对方，否则一方按本合同项下的通讯地址、联系方式发出的信函、文件仍为有效通知，此时接收方迟延收到通知而造成的损失，由该方自行承担。

九、法律适用和争议解决

1. 本合同的订立、效力、解释、履行及争议解决均适用中华人民共和国法律。

2. 所有因本合同引起的或与本合同有关的任何争议，双方应友好协商解决，如果双方在发生争议之日起 30 日内无法通过协商方式解决争议的，则任何一方可将争议提交至甲方所在地有管辖权的人民法院通过诉讼方式解决。

十、其他约定

1. 本合同自甲方法定/授权代表签字并加盖公章且乙方签字之日起生效，有效期至双

扫描全能王 创建

方履行完本合同项下全部义务时止。

2. 本合同一式贰份，甲、乙双方各执壹份，每份具有同等法律效力。

3. 本合同的所有附件（如有）是本合同不可分割的一部分，附件与正文具有同等法律效力，本合同及其附件构成双方就本合同约定的标的达成的完整协议，并取代双方先前与该标的有关的一切口头和书面的洽谈、谈判、通知、备忘录、文件、协议、合同和通讯。

4. 一方未强制执行本合同的一条或若干条款，或未行使其在本合同项下的任何选择权或其他权利，或任何时候未要求另一方履行其在本合同中的任何义务，均不应被理解为该方放弃上述有关条款，或者以任何方式影响本合同的有效性或该方强制执行本合同各项条款的权利，也不应阻止该方在任何时候采取其原本有权采取的其他任何行动。

5. 本合同生效后，除非另有规定，任何一方不得单方面对其进行修改。对本合同的任何修改均应以书面形式作出，并经甲方法定/授权代表签字并加盖公章且乙方签字后方可生效。如果对本合同的任何条款由双方一致同意修改后而与其他条款或附件的任何规定产生冲突、歧义或不一致，应以双方一致同意修改后的条款或附件为准。

6. 无论本合同因任何原因失效、被撤销、解除或终止，本合同中的知识产权、保密、争议解决及适用法律条款继续有效。

7. 无论因何种原因（包括但不限于违反适用的法律法规）导致本合同任何条款完全或部分无效或不具有执行力，不影响本合同任何其他条款的效力、合法和可执行性，双方应当在合法的范围内协商确定新的条款，以保证最大限度地实现原有条款的意图。

8. 本合同未尽事宜，由双方另行协商解决。

（本页以下无正文）

扫描全能王 创建

（本页为签章页）

甲方：中国营养学会（盖章）

法定代表人：

项目负责人：丁昕

甲方盖章：

年 月 日

乙方：【】

签字：

项目负责人：【】

项目依托单位（盖章）：【】

法定代表人：【】

年 月 日

2020.8.26

合同签订地：北京市西城区

扫描全能王 创建
