# Supplementary material for: Relationships among Sleep Time, Physical Activity Time, Screen Time, and Nutrition Literacy of Adolescents: A Cross-Sectional Study in Chongqing, China
Source: Nutrients. 2024 Apr 27;16(9):1314. doi: 10.3390/nu16091314 (PMC11085315; doi:10.3390/nu16091314)
Supplement: Supplementary file 1 [file nutrients-16-01314-s001.zip › Medical research project fund of Chongqing Municipal Health Commission materials.pdf]

编号 KTCX20231428

卫生部医药卫生科技项目
查新咨询报告书

项 目 名 称 高校学生饮食健康行为量表研制及评测

项 目 负 责 人 项江韵　　　　申报级别 厅局级

查 新 性 质 课题查新　　　　学科分类 公共卫生与预防医学

委托单位名称 重庆医科大学公共卫生学院

委 托 人 施雅　　　　电　　话 18487525292

地 址 重庆市渝中区医学院路 1 号

查新单位名称 重庆市卫生健康统计信息中心（国家卫生部定点咨询查新单位）

受 理 人 陈玮嘉　　　　电　　话 023-61965190

地 址 重庆市渝北区宝环路 420 号

委 托 日 期 2023 年 11 月 3 日　　　　报告日期 2023 年 11 月 6 日

中华人民共和国卫生部科教司制

（1997）

一、查新咨询要点（根据《查新咨询委托单》中提供的项目简介和查新咨询要点分析、归纳，并进行具体描述，描述时尽量使用委托人的原用语）：

（1）结合重庆市发布的“吃得文明”倡议，针对大学生群体特征，结合新膳食指南，从“吃得合法、吃得合理、吃得卫生、吃得节约、吃得礼貌”五个维度建立条目库和编制初量表，通过预调查、条目分析、信效度检验等手段最终形成一份科学有效的量表。

（2）利用编制的量表调查高校大学生饮食健康行为的现况，分析其合理用餐、合理膳食、食物浪费、价值观等饮食行为学特征和影响因素，探讨高校大学生饮食存在的问题以及吃方面的文明程度，为打造大学生健康校园、健康食堂提供科学倡导对策。

二、查新咨询要求（根据查新咨询委托人在《查新咨询委托单》中所填查新咨询要求）：

1．通过查新证明在所查范围内国内有无相同或类似研究报道

2．对查新项目的查新点的新颖性进行查证并作出判断

三、检索情况

1． 检索范围：国内

2． 检索工具：见 检索结果四

3． 检索用词：

#1 高校学生

#2 饮食行为

#3 量表

4. 检索策略：

（1）#1 and #2

（2）#1 and #2 and #3

四、检索结果：

1． 检索结果总表

检索工具种类<br>总　　数	检索年限	检索文献<br>总　　量	检出相关<br>文献量	相关<br>文献量
14	20 年	32	8	8

2． 各检索工具检索情况一览表（检索工具名称可用简称或缩写）

检索工具<br>（库/刊等）	检索年限<br>（起止年）
中国生物医学文献数据库（CBM）	1978-Current
北京协和医学院博硕学位论文库（PUMCD）	1981-Current
中国医学科普文献数据库	2000-Current
中国生物医学引文数据库（CBMCI）	1994-Current
西文生物医学文献数据库（WBM）	1978-Current
中国知网学术期刊网络出版总库	1979-Current
CHKD 期刊全文数据库	1994-Current
CHKD 博硕士学位论文全文数据库	1994-Current
CHKD 会议论文、全文数据库	1994-Current
维普中文期刊数据库	1989-Current
万方中国医学数据群（期刊、博硕论文、会议论文、科技成果、专利、标准等）	1998-Current
中华医学会期刊全文数据库	1887-Current
NSTL 外文生物医学文献数据库	1880-Current
燕递（汇医）医学文献服务系统	1800-Current

3． 相关文献目录（采用国家标准著录格式，按相关程度排列，外文题目要译成`中文，将译文括于相应外文文献目录下行）

[1] 王冬.大学生健康生活方式评价量表研制及初步应用研究[D].南方医科大学,2009.
[2] 万雅静,张佳琪,武咏梅.健康传播视域下高校学生的饮食行为倾向[J].现代食品,2022,28(23):221-223.DOI:10.16736/j.cnki.cn41-1434/ts.2022.23.056.
[3] 樊哙.高校体育专业学生饮食行为研究[J].食品研究与开发,2020,41(10):239.
[4] 殷建营,支红霞,李佳佳.周口市高校学生饮食行为及膳食状况调查[J].现代预防医学,2016,43(15):2732-2735.
[5] 王庭瑞,曹玉青,徐生刚.某高校护理专业学生饮食营养状况及健康相关行为调查[J].中国初级卫生保健,2016,30(01):67-69.
[6] 郑荣华,谭斌.某高校医学院学生营养知识、态度、饮食行为及膳食营养状况分析研究[J].中国卫生产业,2015,12(01):173-175+177.DOI:10.16659/j.cnki.1672-5654.2015.01.064.
[7] 宋文波.高校体育专业学生饮食行为习惯调查分析[J].牡丹江师范学院学报(自然科学版),2014(04):53-54.DOI:10.13815/j.cnki.jmtc(ns).2014.04.026.
[8] 孙俊波,汤敏.十堰市 2 所高校学生营养状况、态度及饮食行为调查[J].职业与健康,2013,29(17):2240-2242.DOI:10.13329/j.cnki.zyyjk.2013.17.007.

五、查新咨询结论：

根据委托人的查新要求，采用上述检索策略，检索了 CBM、PUMCD、维普中文期刊数据库、CHKD 期刊全文数据库、万方中国医学数据群等，查得相关文献 8 篇。现将检索结果报告如下:

王冬[1]分析大学阶段是个人逐渐独立和健康行为发展定型的关键阶段，又是接受知识和塑造行为的黄金时期，此时期的生活方式会延续到成年，影响一生的健康。因此基于量化我国大学生健康生活方式，探索影响其健康生活方式养成的因素，而开展本课题研究。 目的： 1.充分考虑我国大陆文化背景、社会结构和价值观，借鉴国外和台湾地区生活方式评价工具，采用系统量表开发技术，编制大学生健康生活方式评价量表，为标准化评估大学生健康生活方式提供客观有效的工具。 2.通过评价量表的应用，了解大学生健康生活方式的现状，初步探索影响大学生健康生活方式的因素，从学校角度提出促使大学生养成健康生活方式的建议对策，为高校倡导大学生养成健康生活方式提供参考。 方法： 1.采用文献研究法、头脑风暴法、焦点小组（大学生）评价法、专家咨询与访谈法编制大学生健康生活方式评价量表条目池。 2.根据专业声誉及研究方向，选取相关领域 39 名专家，采用 Delphi 法对备选条目池进行 3 轮初筛，形成大学生健康生活方式评价量表的初稿。 3.按照地理位置，在东西南北中五个区域各取一所高校，每所高校取样 100 人进行预调查，采用变异系数法、评价指标的应答率、因子分析法、主成分分析法、评价条目间以及评价条目与其维度的相关性分析等方法筛选条目，形成大学生健康生活方式评价量表测试版。 4.按照高校类别抽取 4 所院校在校大学生 2000 人进行现场调查，从信度、效度、反应度等方面对大学生健康生活方式评价量表测试版进行考评。 5.采用专家咨询法，进一步修订测试版，完成大学生健康生活方式评价量表的编制。 6.运用自编大学生健康生活方式调查问卷对两阶段分层抽样抽取的 9 所院校大学生进行现场调查，采用单因素与多因素分析初步探索影响大学生健康生活方式的因素。 结果： 1.大学生健康生活方式评价量表条目池 综合专家和学生对大学生健康生活方式条目重要性评价结果，按预想结构归类成由外显性和内隐性健康生活方式 2 个方面，13 个维度，117 个条目构成的大学生健康生活方式评价量表条目池。 2.大学生健康生活方式评价量表初稿 （1）第一轮征询结果： 专家的积极系数为 84.6%；33 位专家对各条目评价的权威系数的均数在 0.577～0.818 之间，平均为 0.737；专家对各条目重要性判断的总协调系数为 0.212，各维度协调系数在 0.083～0.360；按照重要性转换值均数大小排秩，结合专家对条目修改、合并及删除意见，得出 8 个维度，70 个条目。 （2）第二轮征询结果：

专家的积极系数为93.94%;31位专家对各条目评价的权威系数的均数在0.730～0.945之间，平均为0.834；外显性和内隐性健康生活方式相对重要性评价为0.580：0.420；专家对各条目重要性判断的总协调系数为0.317，各维度协调系数在0.217～0.402；得出外显性和内隐性健康生活方式2个方面，8个维度，44个条目。（3）第三轮征询结果：专家的积极系数为96.77%;30位专家对各条目评价的权威系数的均数在0.778～0.903之间,平均为0.850。外显性和内隐性健康生活方式相对重要性评价为0.583：0.417；专家对各条目重要性判断的总协调系数为0.401，各维度协调系数在0.298～0.526；得出外显性和内隐性健康生活方式2个方面，7个维度，42个条目。 3.大学生健康生活方式评价量表测试版 问卷回收有效率94.4%，经过六种方法对42个初选条目进行了再筛选，筛选出由外显与内隐性健康生活方式2个方面，7个维度，40个条目构成的大学生健康生活方式评价量表测试版。 4.大学生健康生活方式评价量表测试版考评 （1）信度考评结果 大学生健康生活方式评价量表7个维度及外显性、内隐性和总的健康生活方式首次测试得分与间隔二周测试得分相关系数在0.501～0.842之间（$p<0.001$）；总的分半信度相关系数0.865（$p=0.000$），各维度分半信度在0.422～0.746之间;总的Cronbach'α系数为0.898,除健康危害行为维度的Cronbach'α系数为0.417外，其它维度的Cronbach'α系数在0.607至0.910之间。（2）效度考评结果 量表构成能够反映大学生健康生活方式的真实情况，支持内容效度；大学生健康生活方式评价量表测试版总分与WHO生活质量测定量表简表得分总分的相关系数0.571；采用因子分析评价结构效度，KMO统计量为0.932，Bartlett's球形检验结果X2=20883.975（$p=0.000$），适合于因子分析，经因子分析，得到9个公因子，其累计贡献率为54.37%。各因素与量表的设计构想不同，结构效度较不理想，表明本量表需要进一步对条目结构进行修订。 在40个集合效度试验中，成功率为97.5%，在240个区分效度定标试验中，成功率为100%，同时各维度与其它维度间的Spearman相关系数均小于该维度的Cronbach'α系数；各维度得分与总得分间的相关系数均大于各维度得分间的相关系数。各维度分与其分量表分的相关系数均较大，而与其它分量表分相关系数较小，这些均强有力地支持量表的集合和区分效度。（3）反应度考评结果 大学生健康生活方式评价量表测试版各维度首次测试得分与间隔二周测试得分相关系数在0.582～0.843之间（$p<0.05$）；经配对t检验，两次测试结果间除饮食营养行为维度外，其余均无显著性差异（$p>0.05$）；大学生健康生活方式评价量表测试版各维度首次测试得分与间隔四周测试得分相关系数在0.526～0.853之间（$p<0.05$）；经配对t检验，两次测试结果间除运动锻炼行为维度外，其余均无显著性差异

（p>0.05），显示量表的反应度较好。（4）人口统计因素考评结果：大学生健康生活方式评价量表测试版的总分在院校类别、家庭月收入、父母亲文化程度、入学前居住地和是否独生子女等人口统计因素方面存在差异，在性别和年级方面不存在差异。5.大学生健康生活方式评价量表 大学生健康生活方式评价量表由8个维度，38个条目组成，涉及到外显和内隐两个方面生活方式，其中1至22条目组成大学生外显性健康生活方式评定分量表，23至38条目组成大学生内隐性健康生活方式评定分量表。5.大学生健康生活方式评价量表初步应用 （1）大学生健康生活方式评价量表的考核 ①信度 按Spearman-Brown校正，得全量表的分半相关系数为0.840，二个方面的分半相关系数分别为0.710，0.808，8个维度的分半相关系数在0.487～0.737之间；全量表的Cronbach’α系数为0.892，二个方面系数α分别为0.770和0.891，8个维度的α系数在0.518～0.830之间。②效度 KMO统计量为0.929，Bartlett’s球形检验结果X2=54913.124（p=0.000），适合于因子分析。经因子分析，得到9个公因子，累计贡献率为55.02%。除健康危害行为分为两个因子外，其余各条目在因素上的负荷与量表结构吻合，支持结构效度。 在38个集合效度试验中，成功率为97.37%；在259个区分效度定标试验中，成功率为97.30%；各维度与其它维度间的Spearman相关系数均小于该维度的Cronbach’α系数；各维度得分与总得分间的相关系数均大于各维度得分间的相关系数。各维度分与其分量表分的相关系数均较大，而与其它分量表分相关系数较小，这些均支持集合和区分效度。③大学生健康生活方式评价量表的反应度 大学生健康生活方式得分能够区分出健康大学生与患慢性病大学生，经常、一般和很少生病/身体不舒服的大学生。（2）大学生健康生活方式及影响因素初步分析 ①大学生健康生活方式总得分标准分为67，接近中等程度，在各维度上，大学生健康责任行为得分最高，生命欣赏行为次之，其余依序是人际支持行为、生活规律行为、压力管理行为、健康危害行为、饮食营养行为，而运动锻炼行为最低。②性别、气质、自感健康、自感成绩、院校所在地经济水平、院校类别、年级、和谁一起居住、是否学过健康教育与健康促进课程、出生地经济水平、家庭月收入和父母亲文化程度等影响大学生健康生活方式。结论：1.本研究研制的大学生健康生活方式评价量表包含运动锻炼行为、生活规律行为、饮食营养行为、健康危害行为、健康责任行为、人际支持行为、压力管理行为、生命欣赏行为等8个维度，38个条目。2.本量表有较高的信度和效度，可以作为测量大学生健康生活方式的工具。3.大学生健康生活方式接近中等程度，大学生虽然有较高的健康责任，但较少从事运动锻炼行为。4.影响大学生健康生活方式的因素多且复杂，包括生理、心理及认知、学校因素和家庭等多方面的

因素。学校应根据大学生的这些特点采取相应的对策激发不同学生采取健康生活方式的兴趣，促使大学生养成健康生活方式。

万雅静等[2]分析健康是促进人全面发展的必然要求，是经济发展的基础条件，是民族昌盛和国家富强的重要目标，而行为生活方式是影响人类健康的重要因素。本文基于健康传播视域分析了大学生的饮食行为倾向，主要从大学生饮食行为特征、食品安全意识、对食品安全知识的掌握程度 3 方面分析存在的问题，并提出相应的解决对策。

樊哙[3]分析素质教育背景下,我国高校不仅招收文化类学生,还招收艺术体育类学生。体育专业学生因专业特殊性,在校期间往往需同时接受文化教育与体育训练,高强度的脑力活动和体力活动意味着其在饮食摄入更多、营养需求更高,如果体育专业学生无法合理膳食、均衡营养,很可能对身体造成损伤,引起免疫力降低、体能降低等,不利于体育专业学生学习和训练,也不利于学生身心健康成长。《饮食行为学》是美食家(美)玛格丽特·维萨独著的论著,该书主要就人类社会饮食及饮食行为展开了研究,对饮食的发展、饮食的内容、餐桌礼仪均进行了细致描述,是一本关于饮食活动的大百科。

殷建营等[4]了解周口市高校学生饮食行为和营养摄入情况,为制订相应的防控措施提供依据。方法采用称重记账法和回顾法对周口市高校在校就餐学生进行饮食行为和营养状况调查。结果大学生获取营养知识的主要途径是网络和媒体(87.5%);女生吃零食和挑食的比例高于男生(x2=4.396、91.867,P<0.050、0.010),男生添加夜餐的比例高于女生(x2=197.092,P<0.001);存在蛋白质、锌摄入不足,钙、Vit A、Vit B1、Vit B2 缺乏,糖类供能比例高(72.3%),早餐供能比例偏低(19.8%)。结论高校学生获取营养知识的途径少;营养知识普遍欠缺;饮食行为不良、营养失衡、三餐营养分配不合理。应加强教育和指导,改善营养失衡。

王庭瑞等[5]了解护理专业学生饮食营养状况及健康相关行为,为提高学生营养健康水平和身体素质提供科学依据。方法采用调查问卷对学生饮食营养状况及健康相关行为进行调查,采用体质指数评价学生营养状况,应用 x2 检验对调查结果进行分析。结果学生营养不良发生率为 16.44%,营养过剩发生率为 1.73%。学生不仅存在许多不良饮食行为,如不吃早餐、偏食及食用腌制、油炸或烧烤类食品等习惯,而且获取营养知识的途径比较单一,同时还存在一些危害健康的行为,如缺乏体育锻炼、睡眠不足和忌医行为。结论医科类院校应通过多种途径提高学生营养状况和健康水平,为将来更好地开展临床护理工作奠定坚实的基础。

郑荣华等[6]了解该校医学生的行为(P)、态度(A)、营养知识(K)和膳食营养情况的现状,

为日后开展各种医学生营养健康教育活动提供理论和数字依据。方法采用自行设计的营养知识、态度及饮食行为调查问卷,随机整群抽取了石河子大学医学院本科 13 年级 558 名学生进行调查,用 SPSS19.0 软件进行统计分析。随机整群抽取其中 181 名学生,进行了膳食营养调查。结果该校医学生对营养知识掌握有限,平均得分仅为(59.59±14.09),女生高于男生,差异有统计学意义(P<0.05);医学生有较积极的营养态度,96.1%的学生都认为营养对健康很重要;医学生存在一些不合理的饮食行为,有 33.0%的学生不能坚持每天吃早餐;有 29.3%的学生几乎不喝牛奶;一些膳食营养素摄入严重不足,如:钙、维生素 A、维生素 B2 等的摄入量均低于推荐摄入量的 50%。结论医学生学生较缺乏必要的营养知识,饮食行为存在不合理现象,一些营养素摄入严重不足,但对营养的态度积极,应加强对医学生的营养知识教育,纠正不良饮食行为,改善其营养状况。

宋文波[7]对吉林师范大学体育学院大一、大二、大三共 678 名学生的饮食行为习惯进行调查,调查结果表明,学生主食中快餐和小食品占 50.8%,近 1/3 的学生肉类摄入过多,大部分学生不注重副食品对人体营养物质的提供,51.7%的学生能够每天坚持吃早餐,57.8%的学生有边吃饭边喝水或饮料的习惯.建议学校强化体育专业学生平衡膳食、合理营养等知识教育,使学生认识健康饮食的重要性,形成科学的饮食习惯,拒绝不良嗜好,改正不良习惯.

孙俊波等[8]了解十堰市高校大学生营养状况、态度及饮食行为,为开展营养健康教育提供依据。方法采用随机整群抽样方法对该市 2 所高校在校大学生进行问卷调查,采用自行设计的营养状况、态度及饮食行为调查问卷,对 2 所大学问卷结果进行比较分析。结果医学生 489 名,师范生 292 名,共 781 名。781 名学生中营养不良 229 名(29.32%),营养正常 518 例(66.33%),营养过剩 34 例(4.35%);医学生营养不良发生率(31.08%)高于师范生(26.37%),但差异无统计学意义(P>0.05);营养过剩发生率师范生(6.51%)高于医学生(3.07%)(P<0.05)。大多数学生具有较好的营养态度,医学生营养态度明显优于师范生,除"凭借口感选择食物"和"认为现在膳食不合理"2 项问题外,其余问题的回答医学生与师范生比较,差异均有统计学意义(P<0.05)。仅 23.52%的医学生和 8.90%的师范生参加过与营养知识有关的活动。781 名学生中有 28.94%的学生不吃早餐;不吃早餐师范生的比例高于医学生,但差异无统计学意义(P>0.05)。无论是医学生还是师范生不吃早餐的学生较吃早餐的学生更容易发生营养不良(P<0.05)。结论该市高校学生营养不良发生率较高,医学生的营养态度比师范生更积极,但部分大学生饮食行为不佳,不良饮食行为更容易导致营养不良,学校需对学生加强营养健康教育。

上述文献中，[1]采用系统量表开发技术，编制大学生健康生活方式评价量表，为标准化评估大学生健康生活方式提供客观有效的工具，通过评价量表的应用，了解大学生健康生活方式的现状，初步探索影响大学生健康生活方式的因素，从学校角度提出促使大学生养成健康生活方式的建议对策，为高校倡导大学生养成健康生活方式提供参考。[2]基于健康传播视域分析了大学生的饮食行为倾向，主要从大学生饮食行为特征、食品安全意识、对食品安全知识的掌握程度 3 方面分析存在的问题，并提出相应的解决对策。[3]探究高校体育专业学生饮食行为研究。[4]了解周口市高校学生饮食行为和营养摄入情况,为制订相应的防控措施提供依据。[5]了解护理专业学生饮食营养状况及健康相关行为,为提高学生营养健康水平和身体素质提供科学依据。[6]了解该校医学生的行为(P)、态度(A)、营养知识(K)和膳食营养情况的现状,为日后开展各种医学生营养健康教育活动提供理论和数字依据。[7]对吉林师范大学体育学院大一、大二、大三共 678 名学生的饮食行为习惯进行调查。[8]了解十堰市高校大学生营养状况、态度及饮食行为,为开展营养健康教育提供依据。

综上所述，在检索时间、范围内，在国内有采用系统量表开发技术，编制大学生健康生活方式评价量表的研究，在国内有探索高校学生饮食行为的研究，在国内没有从“吃得合法、吃得合理、吃得卫生、吃得节约、吃得礼貌”五个维度建立条目库和编制初量表，调查高校大学生饮食健康行为现况的研究报道。

查 新 人	陈玮嘉	职 称	中级	签字 陈玮嘉
审 核 人	汪应钦	职 称		签字 汪应钦
查新单位	重庆市卫生健康统计信息中心（签章）			2023 年 11 月 6 日

重庆市卫生健康统计信息中心
医药卫生科技查新专用章
