# Supplementary material for: Relationships among Sleep Time, Physical Activity Time, Screen Time, and Nutrition Literacy of Adolescents: A Cross-Sectional Study in Chongqing, China
Source: Nutrients. 2024 Apr 27;16(9):1314. doi: 10.3390/nu16091314 (PMC11085315; doi:10.3390/nu16091314)
Supplement: Supplementary file 1 [file nutrients-16-01314-s001.zip › Table S1-ú¿schedule of survey)Evaluation of nutritional literacy of middle school students in Chongqing.pdf]

Evaluation of nutritional literacy of middle school students in Chongqing

informed consent

Dear Classmates (Freshman, Junior, Senior).

We would like to invite you to participate in the survey on nutritional literacy of secondary school students in Chongqing. With your help, we will collect relevant data to evaluate the nutritional literacy status of secondary school students and provide reference for carrying out relevant nutritional education work. With your help, we will collect relevant data to evaluate the nutritional literacy status of secondary school students and provide reference for carrying out relevant nutritional education work. Please fill out the survey truthfully, we will keep the data confidential and use it only for group analysis without any risk to you. Please fill out the survey truthfully, we will keep the data confidential and use it only for group analysis without any risk to you. This survey is completely voluntary and you can withdraw at any stage.

I have read this informed consent and voluntarily participate in this assessment after full consideration. [Multiple choice]*

○YES

○NO (Please skip to the end of the questionnaire and submit the answer sheet)

PART1, the general situation

1.Your school name is: [Fill in the blank] *

____

2.Your year of birth [multiple choice]*

○1995

○1996

○1997

○1998

○1999

○2000

○2001

○2002

○2003

○2004

○2005

○2006

○2007

○2008

○2009

○2010

3.Your birth month [multiple choice] *

○January

○February

○March

○April

○May

○June

○July

○August

○September

○October

○November

○December

4.Your gender: [multiple choice] *

〇male

〇female

5.What is your nationality: [Multiple choice] *

〇Han nationality

〇Miao nationality

〇Tujia nationality

〇Hui nationality

○Other__________________

6.Your grade: [Multiple choice]*

Grade one of junior high school

〇grade two of junior school

〇Senior One

〇Senior Two

7.Are you currently living on campus: [multiple choice] *

〇YES

○ NO

8.Where your family lives: [Multiple choice]*

〇urban

〇rural

9.Are you an only child?

〇YES

〇NO, How many brothers and sisters __________________ *

10.Your primary guardian (i.e. the person who is primarily responsible for your daily life) : [multiple choice]*

☐Father

☐Mother

Grandparents

☐relative

☐someone else ________________

11.Your parents' level of education: [Matrix multiple choice] *

	Primary and below	junior high school	High school/secondary school/vocational high school	College/Bachelor degree or above	be unaware of
Father.					
Mother					

II. Nutrient Literacy Scale

1.Please sort the layers of the "Dietary Pagoda for Chinese Residents" diagram with the corresponding food groups.

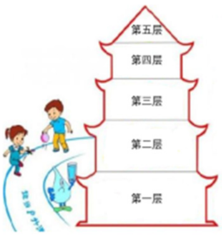

[For sorting questions, please fill in the numbers in parentheses] *

[ ] Pork, chicken, duck, fish, eggs, etc.

[ ] Rice noodles, sweet potatoes, green beans, etc.

[ ] Milk and milk products, soybeans and their products

[ ] Cooking oil, salt

[ ] Vegetables, fruits

2.Based on your judgment, please check the appropriate box [matrix multiple choice question] *

	properly	incorrect	I don't know.
1.Coarse grains (millet, corn, etc.) are more nutritionally complete than fine grains (rice, flour, etc.)			
2.Smoked, salted foods increase cancer risk			
3.Overweight or underweight is associated with an increased risk of disease development			
4.Try to eat lean meat when eating livestock			
5.Good eating habits can prevent chronic diseases like high blood pressure and diabetes			
6.You can exercise less if			

you eat less yourself			
7.Drinking water should be done in small quantities			
8. Meal portioning helps to avoid oral transmission of diseases			

3.The benefits of eating soy products such as tofu and soymilk are [multiple choice] *

It's good for your health.

☐ Good for cardiovascular patients

☐Increase intake of high quality protein

☐ Preventing the negative effects of excessive meat consumption

☐ I don't know.

4.The content of the Dietary Guidelines for Chinese Residents can be easily understood [Single-choice question] *

I've never heard of the Chinese Dietary Guidelines.

I totally disagree.

○Disagree

○ Neutrality

○ Agreed

○I couldn't agree more.

5.When you eat, your first thought is [Single choice] *

○XX Flavors

○Nutrition

○ Fill up on hunger

○Keep in shape

○Other

○No standard

6.When choosing packaged foods, what information on the bag do you look for? [Multiple choice question] *

○Date of manufacture

○Shelf life

○Nutrition Facts Table

○Manufacturer

○I don't know.

○I haven't been paying attention.

7.Please select the 3 snacks you eat most often (snacks: all food and drinks (excluding water) consumed outside of the three meal times of the day) [Multiple Choice] *

☐ Beverages (e.g., cola, yogurt)

☐ Cold drinks (e.g., ice cream, gelato)

☐ Pastries (e.g., bread, cookies)

☐ Confectionery (e.g., chocolate, lollipops)

☐ Fresh fruits and vegetables

☐ Puffed food (e.g. shrimp sticks, snow cakes)

☐ Fried (e.g. potato chips, instant noodles)

☐ Nuts (e.g. melon seeds, peanuts)

☐ Milk and its products (e.g., plain milk, yogurt)

☐ Soybeans and their products (soy milk, dried tofu)

8.Dried fruits and vegetables (e.g. raisins), candied fruits (e.g. dried fruits), canned fruits, etc.

☐ Others __________________

☐ Never eat the above food

9.Recall how many total food items (excluding oils and condiments) you ate <u>during the day yesterday</u>, e.g., a serving of tomato and egg pasta contains 3 food items: tomato, egg, and flour. [Single Choice] *

○0~4 types

○5~8 types

○9~11 types

○≥12 types

○ I don't know.

10.Frequency of breakfast in the past week [multiple choice]*

○I didn't eat it.

○1~2 days

○3~4 days

○5~6 days

○I eat every day.

11.Eating Behavior [Matrix Single Choice] *

	0th	1 time	2 to 6 times	1 time per day	2 or more times per day
1. Frequency of eating fresh fruit in the past week					
2. Frequency of drinking milk, yogurt, soy milk, etc. in the past week					
3. Frequency of drinking sugary beverages (e.g. soft drinks, fruit and vegetable					

juice drinks, tea drinks, functional drinks, etc.) in the past week					
4. Average weekly frequency of having eaten or ordered takeout from Western fast food restaurants (e.g. McDonald's, KFC, pizza restaurants, etc.) in the past month					
5. Average weekly frequency of eating smoked foods (e.g., bacon, sausages, etc.) in the past month					

12.Have you ever had a glass of wine (a glass of wine is the equivalent of half a bottle/glass of beer, a small glass of white wine, a glass of wine or yellow wine) [Single Choice] *

○ Yes

○ No

13.Your last measured height was ___ centimeters (cm) and your weight was ___ kilograms (kg) [fill in the blanks] *

14.Which category do you think your current weight falls into? [Single choice question] *

○Low weight

○Normal

○Overweight

○Obesity

I don't know.

15.Please answer the following questions based on **<u>your last week</u>** (<u>study days</u> are Monday through Friday; <u>days off</u> are Saturday and Sunday)

16.The amount of sleep (including lunch breaks) you get on study days is [single choice] *

○< 7 hours

○[7 , 8) hours

○[8 , 9) hours

○[9 , 10) hours

○≥ 10 hours

17.The amount of sleep (including lunch breaks) you get on your days off is [single choice] *

○<7 hours

○[7 , 8) hours

○[8 , 9) hours

○[9 , 10) hours

○≥ 10 hours

18.The amount of time you spend on physical activity (including housework, leisure activities, sports, and physical exercise such as hiking, jumping rope, etc.) on study days is [single choice] *

○<0.5 hours

○[0.5 , 1) hours

○[1 , 1.5) hours

○[1.5 , 2) hours

○≥ 2 hours

19.The amount of time you spend on physical activity (including housework, leisure activities, sports, and physical exercise such as hiking, jumping rope, etc.) on your days off is [single choice] *

○<0.5 hours

○[0.5 , 1) hours

○[1 , 1.5) hours

○[1.5 , 2) hours

○≥ 2 hours

20.On study days your video screen time (time spent looking at electronic screens such as TV, computers, tablets, video game consoles, or cell phones) is [single choice] *

○<1 hour

○[1 , 2) hours

○[2 , 3) hours

○[3 , 4) hours

○≥ 4 hours

21.On your days off your video screen time (time spent looking at electronic screens such as TV, computers, tablets, video game consoles, or cell phones) is [Single Choice] *

○<1 hour

○[1 , 2) hours

○[2 , 3) hours

○[3 , 4) hours

○≥ 4 hours

22.Acquisition of skills versus comprehension of skills [matrix multiple choice questions] *

	Completely disagree.	disagree	neutral	agree with	couldn't agree more
1. seeks answers to					

questions about nutrition-related knowledge or skills that he or she does not understand					
2. know where to find accurate information when faced with nutrition-related questions or wanting to learn about healthy eating behaviors					
3. have no difficulty finding					

the nutritional informatio n they need from a large number of sources of informatio n					
4. can easily understan d nutritional informatio n on food packages (e.g., energy, protein, sugar, etc.)					
5. can easily understan d health and nutrition recommen					

dations relevant to middle school students					
6. can easily understand nutritional information read in books or on the internet					

23.Interactive skills [matrix radio question] *

	Not at all.	unwilling	neutral	willing (do sth)	very willing
1. Willingness to proactively access nutrition information					
2. Willingne					

ss to share nutritional health knowledge with others					
3. Willingness to receive nutrition education					
4. Willingness to apply nutritional knowledge gained to change poor eating habits					
5. Willingness to persuade others to change bad eating					

habits					

24.Eating Behaviors and Interactive, Critical Skills [Matrix Single Choice] *

	Never had that experience.	never	infrequent	now and then	often	always
1. Replacing vegetables with fruits						
2. Replacing meals with snacks						
3. Replacing fresh vegetables with pickles and sauces						
4. Replacement of fresh fruit with processed fruit						

products such as canned fruit, dried fruit, etc.						
5. Thaw frozen food at room temperature						
6. Will store cooked food at room temperature for more than 2 hours						
7. Separate cutting boards, knives and other utensils for						

processing and serving raw and cooked meat						
8. Try to consume all the meat in bulk and ready-to-eat food in one meal.						
9. Patronizing food stalls such as roadside stands						
10. Have three meals at regular intervals						
11. Talking						

about nutrition topics with others (e.g., friends, family, etc.)						
12. Share with others (e.g., friends, family, etc.) the nutritional information I receive from various sources						
13. Pay attention to nutritional information in the media						

(e.g., school curriculum, Internet, books/brochures, television)						
14. Critical of nutritional information from various sources in society						
15. The accuracy and scientific validity of nutrition-related information in the media can be judged						
16. Be						

able to determine the validity of conflicting nutritional information through media reporting channels.						
17. Will correct their unhealthy behaviors based on nutritional information disseminated by the media						
18. Dare to question deeply entrenched						

sociocultural phenomena related to food and health						

25.Critical Skills [Matrix Multiple Choice] *

	tricky	It's a little difficult.	general	It's a little simple.	very simple
1. Ease of distinguishing whether nutritional information is scientific					
2. Ease of distinguishing between healthy and less healthy foods					
3.					

Ease of assessing the impact of dietary habits on health					
4. When given some advice on healthy eating, I can determine what meets my own health needs					

This is the end of the questionnaire! If you want to know your level of nutritional literacy, please fill in your student number to facilitate our later feedback. [Fill in the blank]

____
